# Assessment of cypermethrin and amitraz resistance and molecular profiling of voltage-gated sodium channel and octopamine tyramine genes of *Rhipicephalus microplus*

**DOI:** 10.3389/fcimb.2023.1176013

**Published:** 2023-05-25

**Authors:** Muhammad Kashif Obaid, Mashal M. Almutairi, Abdulaziz Alouffi, Sher Zaman Safi, Tetsuya Tanaka, Abid Ali

**Affiliations:** ^1^ Department of Zoology, Abdul Wali Khan University Mardan, Mardan, Pakistan; ^2^ Department of Pharmacology and Toxicology, College of Pharmacy, King Saud University, Riyadh, Saudi Arabia; ^3^ King Abdulaziz City for Science and Technology, Riyadh, Saudi Arabia; ^4^ Faculty of Medicine, Bioscience and Nursing, MAHSA University, Jenjarom, Selangor, Malaysia; ^5^ Laboratory of Infectious Diseases, Joint Faculty of Veterinary Medicine, Kagoshima University, Kagoshima, Japan

**Keywords:** ticks, *Rhipicephalus microplus*, acaricide, amitraz, resistance

## Abstract

Control of ticks and tick-borne pathogens is a priority for human and animal health. Livestock-holders extensively rely on acaricide applications for tick control. Different groups of acaricides including cypermethrin and amitraz have been consistently used in Pakistan. There has been a gap in understanding the susceptibility or resistance of *Rhipicephalus microplus*, the most prevalent tick in Pakistan, to acaricides. The present study aimed to molecularly characterize cypermethrin and amitraz targeted genes such as voltage-gated sodium channel (VGSC) and octopamine tyramine (OCT/Tyr) of *R. microplus* ticks in Khyber Pakhtunkhwa (KP), Pakistan to monitor the acaricides resistance. Tick specimens were collected from cattle and buffaloes in northern (Chitral, Shangla, Swat, Dir, and Buner), central (Peshawar, Mardan, Charsadda, Swabi, and Nowshera), and southern districts (Kohat, Karak, Lakki Marwat, Tank, and Dera Ismail Khan) of KP, Pakistan. Different concentrations of commercially available cypermethrin (10%) and amitraz (12.5%) were prepared for *in vitro* larval immersion tests (LIT). In LIT, the average mortality rate of immersed larvae was recorded that was increased gradually with an increase in the concentration of specific acaricide. The larvae’s highest mortality rates (94.5% and 79.5%) were observed at 100-ppm of cypermethrin and amitraz, respectively. A subset of 82 *R. microplus* ticks was subjected to extract genomic DNA, followed by PCR to amplify partial fragments of VGSC (domain-II) and OCT/Tyr genes. The BLAST results of the consensus sequence of VGSC gene (domain-II) showed 100% identity with the acaricides susceptible tick sequence from the United States (reference sequence). Obtained identical sequences of OCT/Tyr genes showed maximum identity (94-100%) with the identical sequences reported from Australia (reference sequence), India, Brazil, Philippines, USA, South Africa, and China. Thirteen single nucleotide polymorphisms (10 synonymous and three non-synonymous) were observed at various positions of partial OCT/Tyr gene fragments. The SNP at position A-22-C (T-8-P) in OCT/Tyr gene has been linked to amitraz resistance in *R. microplus* ticks. Molecular analysis and LIT bioassay’s findings indicate the availability of resistant *R. microplus* ticks in the KP region. To our understanding, this is the first preliminary study to monitor cypermethrin and amitraz resistance via molecular profiling of cypermethrin and amitraz targeted genes (VGSC and OCT/Tyr) in combination with *in vitro* bioassays (LIT) in *R. microplus* ticks from Pakistan.

## Introduction

Ticks are voracious blood sucking chelicerate arthropods that infest all vertebrates except fishes (Dantas-Torres et al., 2012; Guglielmone et al., 2020; Zeb et al., 2022). The direct harms of ticks to livestock and humans include blood loss that causes anaemia, hide damage, irritation, and inflammation. Clinical evidences of inflammation are developed immediately or within a few days after a tick bite, which includes itch, cutaneous rashes, swelling and erythema (Reck et al., 2011; Anderson et al., 2017). However, ticks indirectly act as vectors of various pathogens, including bacteria causing spotted fever rickettsiosis, Lyme disease, anaplasmosis, viruses causing Congo hemorrhagic fever, tick-borne Powassan and encephalitis, and protozoan agents causing theileriosis and babesiosis (Hurtado and Giraldo-Ríos, 2018; Rochlin and Toledo, 2020). In Pakistan, different tick species belonging to various genera such as *Rhipicephalus*, *Hyalomma*, *Haemaphysalis*, *Ixodes*, *Nosomma*, *Amblyomma*, *Ornithodoros*, and *Argus* have been documented. These ticks have been found positive for various pathogens such as *Rickettsia* spp.*, Anaplasma* spp., *Theileria* spp., and *Borrelia* spp. (Karim et al., 2017; Aiman et al., 2022; Khan M. et al., 2022; Khan Z. et al., 2022; Numan et al., 2022).

Livestock-holders extensively rely on various acaricides for the control of ticks and tick-borne pathogens (Obaid et al., 2022). The primary target sites of synthetic pyrethroids (SPs) such as cypermethrin are the voltage-gated sodium channels (VGSCs) (Soderlund, 2012). Structural changes in VGSC protein due to amino acid substitutions may decrease the interaction between SPs and their target sites (Dong, 2007). Molecularly, VGSC gene in *R. microplus* populations to screen the SPs resistance has been extensively studied (Dong et al., 2014). The first detection of single nucleotide polymorphisms (SNPs) in domain-III at position (T-2134-A) of VGSC gene in *R. microplus* ticks was associated with SPs resistance (He et al., 1999). Additionally, various SNPs associated with SPs resistance have been identified in domain-III of VGSC gene of *R. microplus* ticks (Guerrero et al., 2001; Stone et al., 2014; Klafke et al., 2019; Janer et al., 2021). Globally, both synonymous and non-synonymous nucleotide mutations in domain-II of VGSC gene have been recorded in resistant *R. microplus* (Morgan et al., 2009; Stone et al., 2014; Klafke et al., 2019). Amitraz belongs to the formamidine family and can be applied over cattle through spraying and dipping methods, although resistance in *R. microplus* might occur due to the conformational changes in the octopamine tyramine (OCT/Tyr) receptor (Chen et al., 2007; Obaid et al., 2022). Conformational modifications in the octopaminergic receptors due to various SNPs in the OCT/Tyr gene of amitraz-resistant *R. microplus* ticks have been documented (Corley et al., 2013; Baron et al., 2015; Takata et al., 2020).

*Rhipicephalus microplus* is the most prevalent tick in Pakistan (Ali et al., 2019; Ali et al., 2021; Ali et al., 2022b), that acts as a competent vector for numerous pathogens such as *Rickettsia* spp.*, Anaplasma* spp.*, Thelieria* spp., and *Babesia* spp. (Karim et al., 2017; Ali et al., 2021; Alam et al., 2022; Khan Z. et al., 2022). Hence, monitoring the acaricide resistance in the field population of *R. microplus* ticks is essential for the optimal and strategic use of acaricides as well as to slow down the selection of resistance. No study has been conducted to monitor the acaricide resistance via *in vitro* bioassays in combination with molecular profiling of acaricide targeted genes in Pakistan. Hence, this preliminary study aimed to monitor the cypermethrin and amitraz resistance via molecular characterization of the VGSC (domain-II) and OCT/Tyr genes in combination with *in vitro* bioassays of *R. microplus* ticks in Pakistan.

## Materials and methods

### Ethical approval

Ethical approval for this study was taken from the Advanced Studies and Research Board (Dir/A&R/AWKUM/2022/9396) of Abdul Wali Khan University Mardan, Pakistan. Written permission was taken from livestock-holders before the collection of tick specimens. Before collecting ticks, owners were asked about the application of acaricides (cypermethrin and amitraz) over animals’ bodies. Animals were handled carefully as suggested by the “Pakistan’s Prevention of Cruelty to Animal Act” 1890.

### Study area

**Figure 1:**
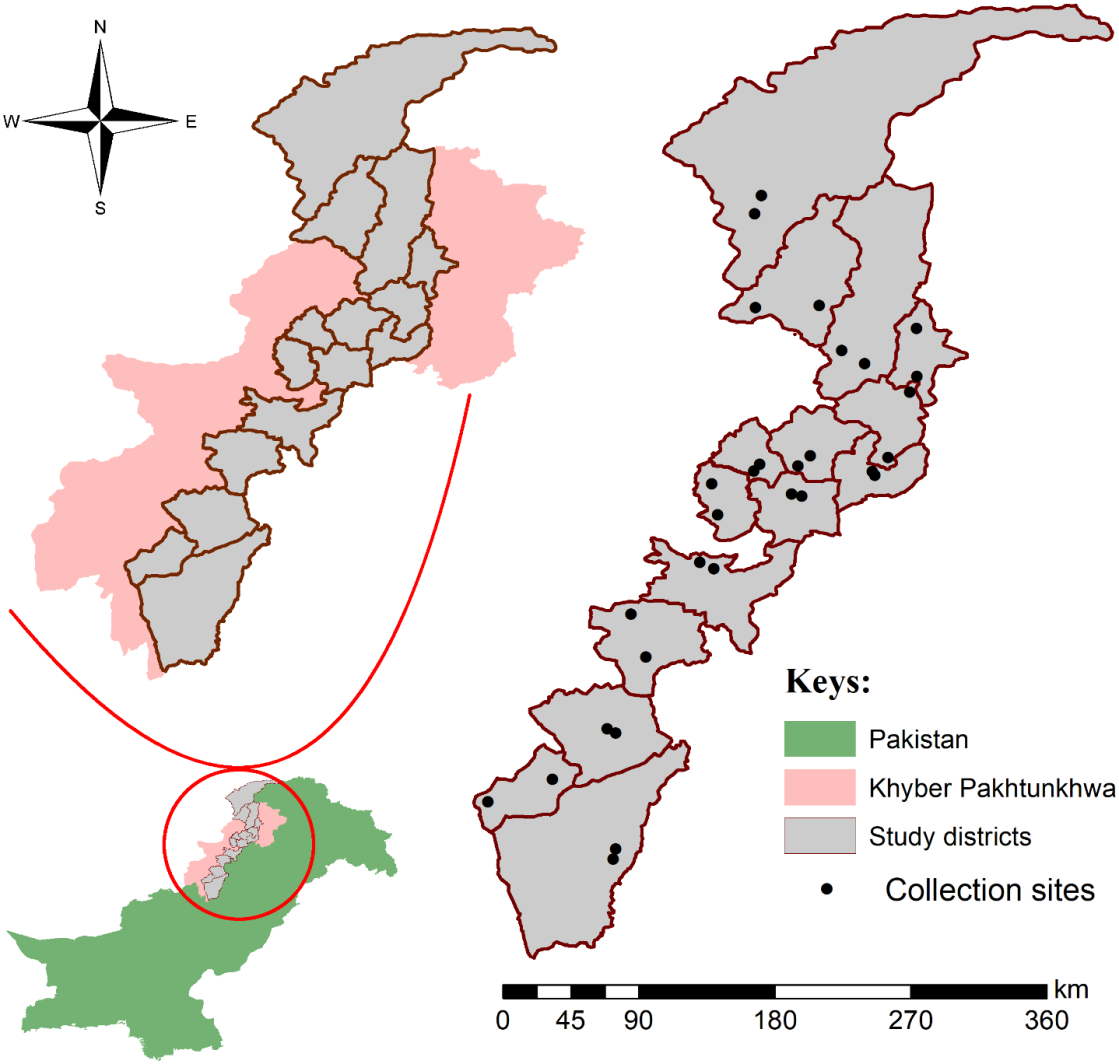
Map showing selected districts and collection sites of ticks.

Khyber Pakhtuknwa province (34°04’50.3”N 71°31’56.6”E) was divided into three main regions including northern, central, and southern; comprised of hilly, plain, and arid areas. Based on easy accessibility such as the availability of transport system, lack of potential security threats, less or no snowfall, and fair weather in the study area, five districts were selected from each region of the study area including northern KP (Chitral, Shangla, Swat, Dir, and Buner), central KP (Peshawar, Mardan, Charsadda, Swabi, and Nowshera), and southern KP (Kohat, Karak, Lakki Marwat, Tank, and Dera Ismail Khan). Localities (number = 30, 2 from each district) at open grazing fields were randomly selected for tick collection. All coordinates were collected through the global positioning system and processed in Microsoft Excel 2013 (Microsoft 365^®^) to design a study map by using ArcGIS 10.3.1 (**Figure 1**).

### Ticks collection, preservation, and morphology-based identification

Partially and fully engorged live ticks were collected from March, 2021 to September, 2021 from cattle and buffaloes in the aforementioned districts (**Figure 1**). Only *R. microplus* ticks including adult (female or male) and nymph stages were identified to species level from the collected specimens based on morphological features under the stereomicroscope (SZ61, Olympus, Japan) using identification keys (Walker et al., 2005; Ali et al., 2022a). Morphologically identified *R. microplus* ticks were categorized into two groups; group one had fully engorged adult female *R. microplus* ticks that were used in LIT bioassays, and ticks in the second group were preserved in 100% ethanol for molecular processing.

### *In vitro* analysis for resistance monitoring

Commercially available cypermethrin (10%) and amitraz (12.5%) were used in LIT bioassays for monitoring acaricide resistance in *R. microplus* ticks. Various dilutions of these acaricides having different concentrations ranging from 5-ppm, 25-ppm, 50-ppm, 75-ppm, and 100-ppm were prepared. After that, 2% (v/v) ethanol and 2% (v/v) Triton™ X-100 in each prepared concentration of 100 ml were added and diluted by using a magnetic stirrer (Food and Agriculture Organization of the United Nations, 2004).

Fully engorged female ticks were divided into three groups on the basis of regions (northern, central, and southern); containing different numbers of ticks (63 northern, 54 central, and 66 southern). Ticks were washed with distilled water, dried on filter paper, and each group was divided and placed in five separate *Petri* dishes for 20 days for oviposition, followed by the larvae hatching in a humidity incubator (BIOBASE, China) at 28 ± 2°C and relative humidity 85 ± 2%. Hatched larvae from each group was divided into two major groups (cypermethrin-group and amitraz-group), and each group was sub-categorized into further two groups (Groups A and B), each containing 500 larvae. Each sub-category A and B was further categorized into five groups, each containing 100 larvae. Standard protocol (Sabatini et al., 2001) was followed in LIT bioassays. In three replicates of LIT bioassays (three separate bioassays for each cypermethrin and amitraz: one from each KP region for each acaricide), the hatched larvae (aged 14–21 days) were treated with various prepared concentrations of cypermethrin and amitraz. Almost 100 larvae from each group (A and B) were immersed for 10 min separately in sepratly prepared concentrations of cypermethrin or amitraz. Distilled water was used as a control-group for all LIT bioassays. Larvae were immediately transferred with a paintbrush to filter papers (Whatman filter papers), dried, and placed in separate labeled *Petri* dishes covered with filter paper. *Petri* dishes were placed in a humidity incubator with the aforementioned specific conditions for 24 hrs. Live and dead larvae were counted through a magnifying lens. The larvae with leg-moving ability were considered alive.

### Genomic DNA extraction

Preserved morphologically identified *R. microplus* ticks including fully engorged adult females and engorged nymphs were washed with distilled water, followed by 70% ethanol to remove secondary contamination, and then immersed in phosphate-buffered saline (pH= 7.4) before the genomic DNA extraction. Sterile needles were used to make holes in the tick’s bodies and incubated at 37°C, and individually whole ticks were cut down into pieces using sterile scissors in separate Eppendorf tubes. Phenol-chloroform method was followed for genomic DNA extraction (Sambrook et al., 1989). DNA quantification was done through NanoQ (Optizen, South Korea), and the extracted DNA was stored at -20°C until further analysis.

### Molecular profiling of targeted genes

Extracted genomic DNA from the same sample was subjected to conventional PCR (GE-96G, BIOER China) for the amplification of both partial fragments of VGSC and OCT/Tyr genes. Two pairs of primers (BmNaF, BmNaR, and OAR-F172, OAR-R587) were used for the amplification of targeted partial fragments of the VGSC (domain-II) and OCT/Tyr genes (**Supplementary Table 1**). A 20 µl PCR reaction mixture was prepared separately for the amplification of each gene, containing 12 µl of Dream*Taq* MasterMix (Thermo Scientific, USA), 4 µl of PCR water, 2 µl of genomic DNA (50-100 ng/µl), and 1 µl of each (forward and reverse) primer. Modified PCR cycling conditions were used to amplify the targeted genes (**Supplementary Table 1**). The PCR amplified products were observed through an agarose gel (2%) in the presence of UV illumination through Gel Doc System (BioDoc-It™ Imaging Systems UVP, LLC).

### DNA purification and sequencing

The PCR amplified products were purified using GeneClean II Kit (Qbiogene) following the manufacturer’s protocol. A total of 162 amplified PCR products (82 for VGSC and 82 for OCT/Tyr genes) were submitted for bidirectional DNA sequencing through the Sanger sequencing method (Macrogen, Inc., Seoul, South Korea). The obtained sequences were trimmed through SeqMan v. 5.00 (DNASTAR), and the primer nucleotide regions as well as poor quality regions were cropped. Trimmed sequences were stored in FASTA format in BioEdit v. 7.0.5 (Hall et al., 2011). One consensus sequence for the VGSC gene while five consensus sequences for the OCT/Tyr gene (one from northern KP, two from each central and southern KP) were obtained from all trimmed sequences through BioEdit and SeqMan v. 5.00. Each consensus sequence was subjected to Basic Local Alignment Search Tool (BLAST) at National Center for Biotechnology Information to find the closest identity with available sequences in GenBank. The closest identity sequences of different countries were downloaded and aligned through ClustalW multiple alignments in BioEdit v. 7.0.5 (Hall et al., 2011). GeneDoc version 2.7 was used to establish the comparison of nucleotides and amino acids (Nicholas et al., 1997).

### Data analyses

The recorded data were analyzed statistically through Microsoft Excel 2016 (Microsoft 365®) and GraphPad Prism v. 5 (GraphPad Software, Inc., San Diego, CA, USA). For the statistical analysis, all the data was inserted into Microsoft Excel 2016. Graphs for *in vitro* analysis were designed through GraphPad Prism v. 5.

## Results

Overall, 301 animal hosts including 161 cattle and 140 buffaloes were examined for tick collection, and 254 out of 301 (84.38%) were found ticks infested, in which cattle (n= 143/254, 56.29%) were highly infested compared to buffaloes (111/254, 43.70%). Among overall 612 collected ticks, 201 (32.84%) were from northern KP, where the highest collection was from district Shangla (48/201, 23.88%), followed by Swat (41/201, 20.40%), Dir (39/201, 19.40%), Buner (37/201, 18.41%), and Chitral (36/201, 17.91%). Ticks collection from central KP was 208/612 (33.99%), including the highest number of ticks from district Mardan (48/208, 23.08%), followed by Charsadda (45/208, 21.63%), Peshawar as well as Swabi (39/208, 18.75%), and Nowshera (37/208, 17.79%). Total ticks from southern KP were 203/612 (33.17%), including the highest numbers from district Lakki Marwat (46/203, 22.66%), followed by D.I. Khan (42/203, 20.69%), Karak (41/203, 20.20%), Tank (39/203, 19.21%), and Kohat (35/203, 17.24%) (**Supplementary Table 2**).

### Morphological identification of *Rhipicephalus microplus*

Overall, 349/612 (57.02%) *R. microplus* ticks were morphologically identified, including adult females (247/349, 70.77%), followed by males (81/349, 23.21%), and nymphs (21/349, 6.02%). Identified ticks from northern KP were 118/349 (33.81%), including the highest numbers of adult females (85/118, 72.03%), followed by males (24/118, 20.34%), and nymphs (9/118, 7.63%). Whereas, a total of 111/349 (31.80%) of *R. microplus* ticks were morphologically identified from the collected specimens of central KP, including adult females (76/111, 68.47%), males (29/111, 26.13%), and nymphs (6/111, 5.41%). Similarly, 120/349 (34.38%) of *R. microplus* ticks were identified that were collected from southern KP, including adult females (86/120, 71.67%), males (28/120, 23.33%), and nymphs (5/120, 4.17%) (**Supplementary Table 2**).

### LIT test findings

Of the 349, almost 183 (52.43%) adult female engorged *R. microplus* ticks were categorized into three groups (northern, central, and southern), and were used in LIT bioassays. Approximately 1100 larvae (500 group-A, 500 group-B, and 100 control-group) were separately treated with different concentrations of cypermethrin or amitraz in each LIT bioassay. Average relevant efficacy of cypermethrin was ranged between 24─94.5% at progressively higher concentrations (5-ppm to 100-ppm) of applied cypermethrin, and the mortality rate of larvae was directly proportional to the applied concentration. Cypermethrin was unsuccessful in causing 100% mortality of larvae even at the highest applied concentration (100-ppm), which indicates the availability of resistant *R. microplus* population in the region.

**Figure 2:**
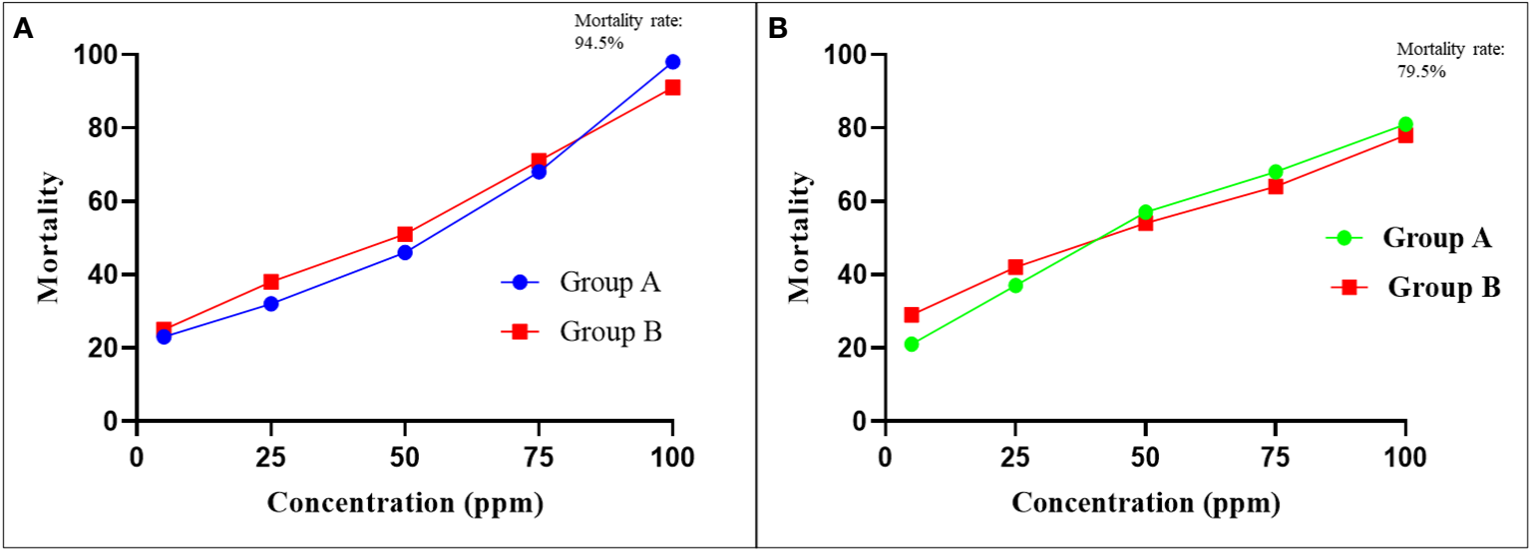
The LIT based average larval mortality of *Rhipicephalus microplus*. **(A)** Plotted mortality curves showing LC_50_ relationship between average larval mortality with respect to applied concentrations of cypermethrin **(B)** Comparative probit mortality curves showing LC_50_ relationship between average larval mortality and acaricide dose (ppm concentrations) for amitraz. The horizontal axis indicates log concentration, while the vertical axis represents mortality.

In the case of amitraz, the immersed larvae’s mortality rate was increased with an increase in applied concentration, and the average recorded mortality rate was 25─79.5%. Hence, the highest mortality rate of larvae was 79.5% at 100-ppm of applied amitraz concentration. Maximum (100%) mortality was not achieved at any applied concentration, indicating the less effectiveness of amitraz against *R. microplus* ticks in the region. Average mortality rate of immersed larvae in LIT bioassays with various concentrations of each cypermethrin and amitraz has been shown in **Figures 2A, B**.

### Molecular and sequence analysis of voltage-gated sodium channel gene (Domain-II)

Genomic DNA from *R. microplus* ticks (82/349, 23.50%) was extracted, including from northern KP (27/82, 32.92%), central KP (28/82, 34.15%), and southern KP (27/82, 32.92%), that were molecularly processed for the characterization of cypermethrin and amitraz targeted genes (VGSC and OCT/Tyr) (**Supplementary Table 2**).

**Figure 3:**
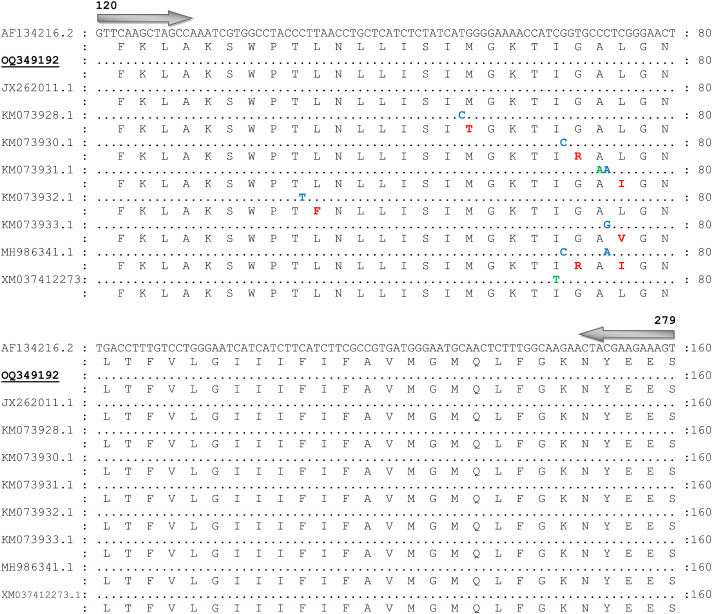
Nucleotide sequence and translated amino acid sequence alignment of the fragment of voltage-gated sodium channel gene (domain-II) of *Rhiphicephalus microplus* with the acaricide susceptible tick reference sequence (GenBank Accession No. AF134216). Similar nucleotides are marked with dots. Non-synonymous single nucleotide polymorphisms, synonymous SNPs, and amino acid substitutions are represented with blue, green, and red colors, respectively. Present sequence is labeled with bold and underlined and GenBank accession number OQ349192.

Consensus sequence (OQ349192) obtained from 82 trimmed sequences of the partial fragments of VGSC gene (domain-II) was 160 bp. The BLAST results of the obtained consensus sequence showed the closest identity (100%) with the acaricide susceptible tick sequence reported from the United States (AF134216.1), which was used as a reference sequence for comparative analysis. Other identical sequences used in the comparative alignment of VGSC gene (domain-II) were reported from India (JX262011.1; 100% identity), (JQ693152.1; 99.38% identity), Mexico (KM073928.1; 99.38% identity), (KM073930.1; 99.38% identity), (KM073931.1; 99.75% identity), (KM073932.1; 99.38% identity), (KM073933.1; 99.38% identity), (MH986341.1; 99.75% identity), and China (XM-037412273.1; 99.38% identity). No single nucleotide polymorphism was observed in the consensus sequence of VGSC gene (domain-II) with the acaricide susceptible tick reference sequence (AF134216.1) (**Figure 3**).

### Sequence analysis of partial fragment of octopamine tyramine gene

Among all 82 trimmed sequences of OCT/Tyr gene, five identical consensus sequences as northern KP (OQ473125; 342 bp), central KP 1 (OQ511314; 288 bp), central KP 2 (OQ397121; 342 bp), southern KP 1 (OQ442834; 342 bp), and southern KP 2 (OQ454517; 342 bp) were obtained. The BLAST results of trimmed sequences showed the highest identity (94-100%) with available sequences reported from Australia (AJ010743.1), India (MN062624.1, MG490863.1, MG551470.1), Brazil (MW218470.1), Philippines (LC604794.1), USA (EF490687.1, EF490688.1), South Africa (KR081351.1, KR081352.1, KR081353.1), and China (XM_037420463.1).

**Figure 4:**
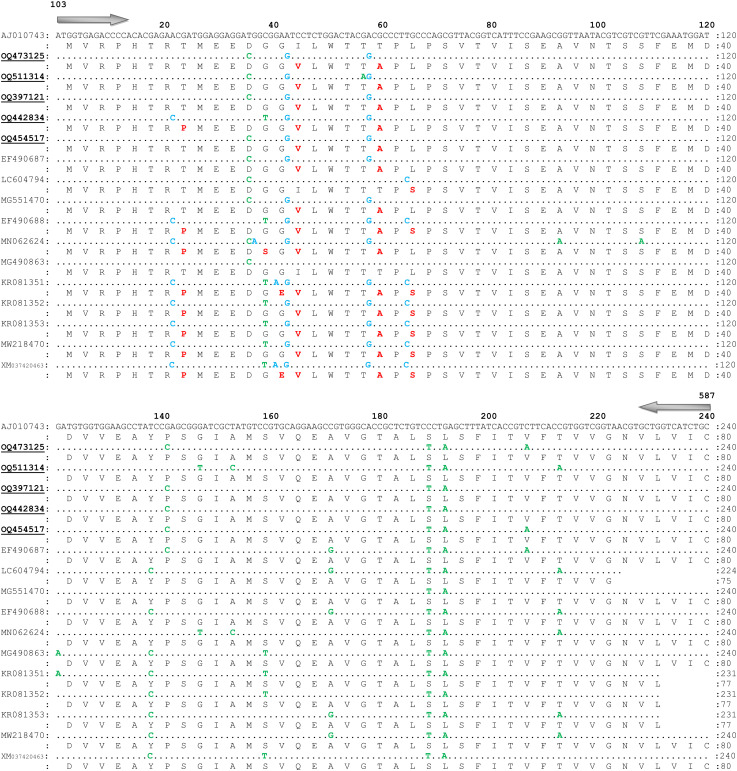
Nucleotide sequence and translated amino acid sequence alignment of the fragment of octopamine tyramine gene of *Rhiphicephalus microplus* with reference sequence (GenBank Accession No. AJ010743.1). Similar nucleotides are marked with dots. Non-synonymous single nucleotide polymorphisms, synonymous SNPs, and amino acid substitutions are shown by blue, green, and red colors, respectively. Present sequences are labeled with bold and underlined, and submitted to the GenBank under accession numbers; northern KP (OQ473125), central KP 1 (OQ511314), central KP 2 (OQ397121), southern KP 1 (OQ442834), and southern KP 2 (OQ454517).

Overall, 13 SNPs (10 synonymous and three non-synonymous) at various positions in the coding region of the partial fragment of OCT/Tyr gene were detected. Among them, three non-synonymous SNPs resulted into amino acid substitutions while the remaining SNPs had no effect on amino acid substitution (**Supplementary Table 3**; **Figure 4**). Additionally, one SNP (non-synonymous) at position A-22-C was observed in the sequence (OQ442834), which was only available in previously reported sequences from resistant ticks (EF490688.1, MN062624.1, KR081351.1, KR081352.1, KR081353.1, MW218470.1, and XM_037420463.1). Three synonymous SNPs (C-39-T, G-147-T, and T-153-C) were identified in the obtained sequences that were available in the reported sequences from resistant ticks (**Supplementary Table 3**; **Figure 4**).

## Discussion

Selection of resistance to various acaricides has been recorded globally, although direct application of acaricides to animal hosts is a common practice for tick control. In Pakistan, resistance to different acaricides has been documented through adult immersion test (AIT) and LIT bioassays (Sajid et al., 2009; Kamran et al., 2021). However, a sufficient mortality rate due to the fipronil and ivermectin was not achieved previously, providing evidences regarding the resistant *R. microplus* population in Pakistan (Sajid et al., 2009; Kamran et al., 2021). Our preliminary findings based on LIT bioassays unveiled the resistant *R. microplus* ticks to commercially available cypermethrin and amitraz, because both acaricides failed to cause the expected mortality of larvae. The LIT bioassay-based based results were supported by molecular analysis, as the identification of various SNPs in the OCT/Tyr gene (specifically A-22-C) revealed amitraz resistant *R. microplus* ticks. Herein, cypermethrin and amitraz resistance was monitored via molecular characterization of the VGSC (domain-II) and OCT/Tyr genes in combination with *in vitro* bioassays (LIT) in *R. microplus* population for the first time in Pakistan.

In tick’s neurons, VGSC is responsible for generating a membrane potential, that has four domains (I, II, III, and IV) and codes for the membrane protein. However, only domain-II and III have been associated with resistance to SPs in *R. microplus*. In this study, we have not identified any single SNP in the consensus sequence of VGSC gene (domain-II). Various studies have linked different non-synonymous SNPs identified in VGSC gene (domain-II) of *R. microplus* reported from India (C-190-A), Mexico (C-148-T), (T170-C), (G-184-C), (C-190-G), (G-184-C, C-190-A). Additionally, different SNPs in domain-III (C-2130-T, T-2134-A; F-712-I, and C-2136-A; F-712-L) of VGSC gene of *R. microplus* have been documented and associated with resistance to SPs (Morgan et al., 2009; Domingues et al., 2012; Lovis et al., 2012; Stone et al., 2014; Robbertse et al., 2016; Klafke et al., 2019; Janer et al., 2021). All of these non-synonymous SNPs resulted in amino acid substitutions that caused modifications in the VGSC gene of *R. microplus* ticks.

The target site of amitraz is OCT/Tyr receptor in *R. microplus* ticks. Altogether 37 SNPs were identified previously in OCT/Tyr gene reported from susceptible and resistant *R. microplus* ticks, in which two non-synonymous SNPs (A-22-C; T-8-P and T-65-C; L-22-S) were linked with the amitraz resistance (Chen et al., 2007), that resulted in the phenotypic modification of OCT/Tyr receptor. These two non-synonymous SNPs were also revealed at positions A-155-C (T-8-P) and T-200-C (L-22-S) of the amitraz targeted site (Baron et al., 2015; Sungirai et al., 2018). Additionally, Baron et al. (2015) also associated these two SNPs with amitraz resistance, and confirmed this association via larval packet test (LPT) as all survived larvae (after LPT) were resistant to amitraz. Present study observed synonymous and non-synonymous SNPs in the obtained sequences, that have been previously reported in the susceptible (EF490687.1, MG551470.1, and LC604794.1) and resistant (MN062624.1, MG490863.1, MW218470.1, EF490688.1, KR081351.1, KR081352.1, KR081353.1, and XM_037420463.1) *R. microplus* ticks. Only a single SNP at position A-22-C (T-8-P) was observed in the coding region of OCT/Tyr sequence (OQ442834), that may play a role in the selection of amitraz resistance in *R. microplus* ticks (Baron et al., 2015). These findings suggest that the population of resistant *R. microplus* ticks to cypermethrin and amitraz has been developed in KP, thus, the livestock-holders must be educated regarding the usage of these drugs. Since only domain-II of VGSC gene was molecularly characterized, this limits the findings of the present study regarding the exact status of acaricide resistance. Therefore, domain-III of VGSC and other resistance associated genes should be sequenced through proper surveillance studies, to conclude about the exact status of acaricide resistance in the surveyed region.

## Conclusion

*Rhipicephalus microplus* ticks from the survey regions showed marked resistance to cypermethrin and amitraz, that was confirmed through *in vitro* bioassays and molecular analyses. Cypermethrin caused a higher mortality rate of *R. microplus* larvae than amitraz. To our knowledge, this is the first preliminary molecular approach regarding the two acaricide targeted genes (VGSC and OCT/Tyr) in Pakistan. There is a need to further explore the domain-II and III of VGSC and other acaricide targeted genes of *R. microplus* to conclude about the resistance status. To acquire knowledge about acaricide resistance, it is critical to carry out periodic surveillance regarding the selection of resistance. This study will be helpful for the government as well as local bodies to define effective tick control strategies and sensible usage of acaricides.

## Data availability statement

The datasets presented in this study can be found in online repositories. The names of the repository/repositories and accession number(s) can be found below: https://www.ncbi.nlm.nih.gov/genbank/, OQ473125, OQ511314, OQ397121, OQ442834, OQ454517, OQ349192.

## Ethics statement

Ethical approval for this study was taken from the Advanced Studies and Research Board (Dir/A&R/AWKUM/2022/9396) of Abdul Wali Khan University Mardan, Pakistan. Written permission was taken from livestock-holders before the collection of tick specimens.

## Author contributions

AAli, AAlo, MMA, TT, MKO and SZS carried out the experimental design of the study. AAli, MKO, collected the tick samples. AAli, AAlo, MMA, TT, MKO and SZS performed the experiments. AAli and MKO performed the sequences and statistical analysis. All authors contributed to the article and approved the submitted version.
