# Supplementary material for: Assessment of cypermethrin and amitraz resistance and molecular profiling of voltage-gated sodium channel and octopamine tyramine genes of Rhipicephalus microplus
Source: Front Cell Infect Microbiol. 2023 May 25;13:1176013. doi: 10.3389/fcimb.2023.1176013 (PMC10248163; doi:10.3389/fcimb.2023.1176013)
Supplement: Supplementary file 1 [file DataSheet_1.docx]

Supplementary Material

**Assessment of Cypermethrin and Amitraz Resistance and Molecular Profiling of Voltage-Gated Sodium Channel and Octopamine Tyramine Genes of *Rhipicephalus microplus***

Muhammad Kashif Obaid^1^, Mashal M. Almutairi^2^, Abdulaziz Alouffi^3^, Sher Zaman Safi^4^, Tetsuya Tanaka^5*^, Abid Ali^1*^

*** Correspondence:** ^*^Abid Ali; [uop_ali@yahoo.com](mailto:uop_ali@yahoo.com) , ^*^Tetsuya Tanaka; [k6199431@kadai.jp](mailto:k6199431@kadai.jp)

**Table 1.** List of primers used to amplify the partial fragments of the voltage-gated sodium channel and octopamine tyramine genes.

| **Tick Specie** | **Targeted Genes** | **Primer Sequence (5'-3')** | **PCR Conditions** | **Amplicon Size** | **Study References** |
| --- | --- | --- | --- | --- | --- |
| *Rhipicephalus microplus* | VGSC genes | **BmNaF5:**  TAC GTG TGT TCA AGC TAG C  **BmNaR5:**  ACT TTC TTC GTA GTT CTT GC | 95ºC─3 min, 40X (95°C─30 sec, 56°C─30 sec, 72°C─30 sec), 72°C─7 min | 167 bp | Morgan et al., 2009 |
| *Rhipicephalus microplus* | OCT/Tyr genes | **OAR-F172:**  AGC ATT CTG CGG TTT TCT AC  **OAR-R587:**  GCA GAT GAC CAG CAC GTT ACC G | 94ºC─3 min, 40X (94°C─30 sec, 54°C─45 sec, 72°C─1 min), 72°C-7 min | 394 bp | Singh et al., 2021 |

**Table 2.** Rhythmic view of study; survey regions, morphologically identification of *Rhipicephalus microplus* and their molecular analysis.

| **KP Regions** | **Districts** | **Hosts** | | | | **Collected Ticks (%)** | **Ticks** | | | | **Female Ticks Used for *In Vitro* Tests** | **DNA Extraction** | | | **Subjected to PCR** | **Sequencing** (for VGSC genes) | **Sequencing** (for OCT/Tyr genes) |
| --- | --- | --- | --- | --- | --- | --- | --- | --- | --- | --- | --- | --- | --- | --- | --- | --- | --- |
|  |  | **Examined** | | **Infested** | |  | **Stages** | | | |  |  |  |  |  |  |  |
|  |  | **Cattle** | **Buffaloes** | **Cattle** | **Buffaloes** |  | **Male** | **Female** | **Nymph** | **Total** |  | **Female** | **Nymph** | **Total** |  |  |  |
| **Northern** | Chitral | 9 | 8 | 7 | 6 | 36 (17.91) | 5 | 16 | 3 | 24 | 10 | 7 | 1 | 8 | 8 | 8 | 8 |
|  | Shangla | 13 | 11 | 12 | 7 | 48 (23.88) | 6 | 19 | 2 | 27 | 15 | 4 | 1 | 5 | 5 | 5 | 5 |
|  | Swat | 9 | 9 | 8 | 6 | 41 (20.40) | 4 | 17 | 2 | 23 | 13 | 4 | - | 4 | 4 | 4 | 4 |
|  | Dir | 10 | 10 | 9 | 8 | 39 (19.40) | 4 | 15 | - | 19 | 10 | 6 | - | 6 | 6 | 6 | 6 |
|  | Buner | 11 | 8 | 10 | 9 | 37 (18.41) | 5 | 18 | 2 | 25 | 15 | 3 | 1 | 4 | 4 | 4 | 4 |
| **Northern (total%)** | | **52** | **46** | **46** | **36** | **201** (32.84) | **24** (20.34) | **85** (72.03) | **9 (**7.63**)** | **118** (33.81) | **63** (34.43) | **24** (88.89) | **3** (11.11) | **27 (**32.92) | **27 (**32.92) | **27 (**32.92) | **27 (**32.92) |
| **Central** | Peshawar | 13 | 10 | 12 | 7 | 39 (18.75) | 6 | 16 | 1 | 23 | 10 | 7 | 1 | 8 | 8 | 8 | 8 |
|  | Mardan | 10 | 8 | 9 | 6 | 48 (23.08) | 5 | 19 | 2 | 26 | 13 | 6 | 1 | 7 | 7 | 7 | 7 |
|  | Charsadda | 12 | 11 | 11 | 8 | 45 (21.63) | 7 | 14 | - | 21 | 10 | 4 | - | 4 | 4 | 4 | 4 |
|  | Swabi | 11 | 10 | 10 | 9 | 39 (18.75) | 5 | 13 | 1 | 19 | 9 | 5 | - | 5 | 5 | 5 | 5 |
|  | Nowshera | 8 | 9 | 7 | 8 | 37 (17.79) | 6 | 14 | 2 | 22 | 12 | 3 | 1 | 4 | 4 | 4 | 4 |
| **Central (total%)** | | **54** | **48** | **49** | **38** | **208** (33.99) | **29** (26.13) | **76** (68.47) | **6 (5.41)** | **111** (31.80) | **54** (29.51) | **25** (92.59) | **3** (10.71) | **28 (**34.15) | **28 (**34.15) | **28 (**34.15) | **28 (**34.15) |
| **Southern** | Kohat | 10 | 7 | 8 | 6 | 35 (17.24) | 7 | 17 | - | 24 | 13 | 5 | - | 5 | 5 | 5 | 5 |
|  | Karak | 8 | 9 | 7 | 7 | 41 (20.20) | 6 | 14 | 1 | 21 | 11 | 3 | - | 3 | 3 | 3 | 3 |
|  | Lakki Marwat | 14 | 11 | 12 | 8 | 46 (22.66) | 7 | 17 | 1 | 25 | 11 | 6 | 2 | 8 | 8 | 8 | 8 |
|  | Tank | 12 | 10 | 11 | 9 | 39 (19.21) | 3 | 21 | 3 | 27 | 17 | 5 | 1 | 6 | 6 | 6 | 6 |
|  | Dera Ismail Khan | 11 | 9 | 10 | 7 | 42 (20.69) | 5 | 17 | 1 | 23 | 14 | 4 | 1 | 5 | 5 | 5 | 5 |
| **Southern (total%)** | | **55** | **46** | **48** | **37** | **203** (33.17) | **28** (23.33) | **86** (71.67) | **5 (**4.17) | **120** (34.38) | **66** (36.06) | **23** (85.19) | **4** (14.81) | **27 (**32.92) | **27 (**32.92) | **27 (**32.92) | **27 (**32.92) |
| **Total (%)** | | **161** (54.49) | **140** (46.51) | **143** (56.29) | **111** (43.70) | **612** | **81** (23.21) | **247** (70.77) | **21** (6.02) | **349** (57.02) | **183** (52.43) | **72** (87.80) | **10** (12.19) | **82** (23.50) | **82** (100.00) | **82** (100.00) | **82** (100.00) |
|  |  | **301** | | **254** (84.38) | |  |  |  |  |  |  |  |  |  |  |  |  |

**Table 3.** Identified single nucleotide polymorphisms in the obtained sequences of the partial fragments of octopamine tyramine genes of *Rhipicephalus microplus*

| **SNPs** | **NS/S** | **Amino Acid Substitutions** | **Detected in Khyber Pakhtunkhwa (KP)** | | | | | **Detected in Susceptible and Resistant Previously** | |
| --- | --- | --- | --- | --- | --- | --- | --- | --- | --- |
|  |  |  | **N. KP**  **(**OQ473125**)** | **C. KP 1**  (OQ511314) | **C. KP 2** (OQ397121) | **S. KP 1 (**OQ442834**)** | **S. KP 2 (**OQ454517 **)** | **Susceptible** | **Resistant** |
| A-22-C | NS | T-8-P | **─** | **─** | **─** | Yes | **─** | **─** | Yes |
| T-36-C | S | **─** | Yes | Yes | Yes | Yes | Yes | Yes | Yes |
| C-39-T | S | **─** | **─** | **─** | **─** | Yes | **─** | **─** | Yes |
| A-43-G | NS | I-15-V | Yes | Yes | Yes | Yes | Yes | Yes | Yes |
| G-57-A | S | **─** | **─** | Yes | **─** | **─** | **─** | **─** | **─** |
| A-58-G | NS | T-20-A | Yes | Yes | Yes | Yes | Yes | Yes | Yes |
| G-141-C | S | **─** | Yes | **─** | Yes | Yes | Yes | Yes | **─** |
| G-147-T | S | **─** | **─** | Yes | **─** | **─** | **─** | **─** | Yes |
| T-153-C | S | **─** | **─** | Yes | **─** | **─** | **─** | **─** | Yes |
| C-189-T | S | **─** | Yes | Yes | Yes | Yes | Yes | Yes | Yes |
| C-192-A | S | **─** | Yes | Yes | Yes | Yes | Yes | Yes | Yes |
| C-207-A | S | **─** | Yes | **─** | **─** | **─** | Yes | Yes | **─** |
| C-213-A | S | **─** | **─** | Yes | **─** | **─** | **─** | Yes | Yes |

**NS**; non-synonymous, **S**; synonymous, **N**; northern, **C**; central, **S**; southern

**Reference**

Morgan, J. A., Corley, S. W., Jackson, L. A., Lew-Tabor, A. E., Moolhuijzen, P. M., and Jonsson, N. N. (2009). Identiﬁcation of a mutation in the para-sodium channel gene of the cattle tick *Rhipicephalus (Boophilus) microplus* associated with resistance to synthetic pyrethroid acaricides. Int. J. Parasitol. 39 (7), 775–779. doi: 10.1016/j.ijpara.2008.12.006

Singh, N. K., Singh, H., Singh, N. K., and Rath, S. S. (2021). Genotyping Amitraz resistance proﬁles in *Rhipicephalus microplus* canestrini (Acari: ixodidae) ticks from punjab, India. Ti*cks.Tick. Borne. Dis.* 12, 101578. doi: 10.1016/j.ttbdis.2020.101578
